# Ultrasonography for the Prediction of High-Volume Lymph Node Metastases in Papillary Thyroid Carcinoma: Should Surgeons Believe Ultrasound Results?

**DOI:** 10.1007/s00268-020-05755-0

**Published:** 2020-09-11

**Authors:** Chunhao Liu, Lei Zhang, Yuewu Liu, Yu Xia, Yue Cao, Ziwen Liu, Ge Chen, Ning Liu, Zhonghua Shang, Jinbao Yang, Qinghe Sun, Xiaoyi Li

**Affiliations:** 1 Department of General Surgery, Peking Union Medical College Hospital, Chinese Academy of Medical Sciences and Peking Union Medical College, No. 1 Shuai Fu Yuan, Dongcheng District, Beijing, 100730 China; 2 Department of Liver Surgery, Peking Union Medical College Hospital, Chinese Academy of Medical Sciences and Peking Union Medical College, Beijing, 100730 China; 3 Department of Ultrasound, Peking Union Medical College Hospital, Chinese Academy of Medical Sciences and Peking Union Medical College, Beijing, 100730 China; 4 McKusick-Zhang Center for Genetic Medicine, State Key Laboratory of Medical Molecular Biology, Institute of Basic Medical Sciences, Chinese Academy of Medical Sciences and Peking Union Medical College, Beijing, 100730 China; 5 Department of General Surgery, Second Hospital of Shanxi Medical University, Taiyuan, 030001 Shanxi Province China; 6 Department of General Surgery, People’s Liberation Army Bethune International Peace Hospital, Shijiazhuang, 050082 Hebei Province China; 7 Department of General Surgery, Cangzhou People’s Hospital, Cangzhou, 061000 Hebei Province China

## Abstract

**Background:**

Lymph node metastasis (LNM) often occurs in papillary thyroid carcinoma (PTC); the efficacy of ultrasound for predicting high-volume lymph node metastases (LNMs) in patients with PTC remains unexplored.

**Methods:**

The medical records of 2073 consecutive PTC patients were reviewed. Sensitivity, specificity, positive predictive value (PPV) and negative predictive value (NPV) were calculated to evaluate the efficacy of ultrasound. Risk factors for LNM/high-volume LNMs and lymph node involvement on ultrasound (usLNM) were identified by univariate and multivariate analyses.

**Results:**

Of all the patients, 936 (45.2%) patients had LNMs, and 254 (12.3%) patients had high-volume LNMs. The sensitivity of ultrasound for detecting LNM/high-volume LNMs was 27.9% and 63.8%, respectively; the specificity was 93.1% and 90.3%, respectively. The NPV for ultrasound in detecting high-volume LNMs was 94.7%. In multivariate analysis, male sex (OR = 2.108, *p *< 0.001), tumor diameter > 1.0 cm (OR = 2.304, *p *< 0.001) and usLNM (+) (OR = 12.553, *p *< 0.001) were independent clinical risk factors for high-volume LNMs. Tumor diameter > 1 cm (OR = 3.036, *p* < 0.001) and male sex (OR = 1.642, *p *< 0.001) were independent clinical risk factors for usLNM; a skilled sonographer (OR = 1.121, *p *= 0.358) was not significantly associated with usLNM.

**Conclusions:**

Lymph node involvement found by ultrasound has great predictive value for high-volume LNMs; the NPV is very high for patients without lymph node involvement on ultrasound. The ultrasound results do not appear to be influenced by the experience of the sonographer.

**Electronic supplementary material:**

The online version of this article (10.1007/s00268-020-05755-0) contains supplementary material, which is available to authorized users.

## Introduction

Papillary thyroid carcinoma (PTC) accounts for approximately 85% of thyroid malignancies. The incidence has been increasing rapidly in recent years, and it has even become the highest of all malignancies in Korea [1–3]. With appropriate treatment, the prognosis of PTC is satisfactory, and the 10-year overall survival rate is over 90% [4]. Throughout the lifespan, the main problems confronted by most patients are recurrence and reoperation.

Cervical lymph node metastases (LNMs) are very common, especially in the central compartment, and the metastatic rate is 20–90% [5]. The prognostic significance and surgical management of LNM remain controversial. However, several studies have shown that LNM is the second independent risk factor for PTC patients following distant metastasis. Moreover, for patients with high-volume LNMs (>5 pathological lymph node metastases), the risk of recurrence increases significantly [6, 7]. Based on the 2015 ATA risk stratification system, patients with high-volume LNMs have an intermediate risk of recurrence, and treatment for these patients should thus be highly proactive [8]. Therefore, identifying patients with high-volume LNMs before surgery is of great significance for treatment decision-making.

Ultrasound is the most convenient and effective method for screening thyroid disease. However, ultrasound is operator dependent and cannot image deep anatomic structures that are adequately shadowed by bone or air. Although studies have noted that the efficacy of lymph node metastasis detection by ultrasound is not ideal [9, 10], the preoperative detection of high-volume LNMs by ultrasound remains unexplored. This study was designed to evaluate the value of preoperative ultrasound for predicting high-volume LNMs in patients with papillary thyroid carcinoma.

## Patients and methods

This retrospective study included a total of 2073 consecutive PTC patients (male: 490, female: 1583) who underwent primary surgery in our institution for 1 year. This study was approved by the Ethics Committees of Peking Union Medical College Hospital, and all the patients had signed the informed consent.

The inclusion criteria were as follows: (1) newly diagnosed PTC and (2) underwent lobectomy or near-total/total thyroidectomy with cervical lymph node dissection (LND) according to the Chinese guidelines (All PTC patients should undergo at least ipsilateral central neck dissection and lateral neck dissection when cN1b was indicated. The recommendation for primary thyroid surgery is similar to ATA guidelines) [11]. The exclusion criteria were as follows: (1) underwent revision surgery; (2) not underwent cervical LND; and (3) non-PTC on pathological examination. In multifocal cases, the largest diameter of tumors was included for analysis. The number of metastatic lymph nodes was calculated by pathological examination. More than 5 metastatic lymph nodes were defined as high-volume LNMs.

Preoperative ultrasound, which was performed in all patients, provided data on the extent of thyroid disease and suspicious cervical central and lateral lymphadenopathy. There are six features which were used for judging clinically involved nodes (cN1) in our institution. They are enlargement and round shape, loss of the fatty hilum, peripheral vascularity, hyperechogenicity, cystic aspect and microcalcifications. The criteria for lymph nodes involvement are any two or more of the first three features, or any one or more of the last three features, when chronic lymphocytic thyroiditis was considered on ultrasound examination; the criteria for lymph nodes involvement are any one or more of the six features when chronic lymphocytic thyroiditis wasn’t considered. In terms of analyzing the influence of the sonographer on the examination results, sonographers were divided into two groups based on their experience: group A, general sonographer who had less than 5 years experience in thyroid ultrasound with approximately 600–700 cases per year, and group B, skilled thyroid sonographer who had performed over 1000 thyroid screening cases per year over the past 5 years.

In this study, we compared the preoperative ultrasound results and postoperative pathological LNM to determine the accuracy of the ultrasound diagnosis of lymph node metastasis. The ultrasound findings of lymph node involvement as a criterion for suggesting high-volume LNMs were used to evaluate the efficacy of ultrasound diagnosis for high-volume lymph node metastases. We also analyzed the clinical factors associated with pathological lymph node metastasis, high-volume lymph node metastases and lymph node involvement on ultrasound.

Statistical analysis was performed using SPSS software (version 25.0). For sensitivity and specificity evaluations, the following formulas were applied using the histopathological report as the gold standard: sensitivity = true positives/(true positives + false negatives) and specificity = true negatives/(true negatives + false positives). Positive predictive value = true positives/(true positives + false positives) and negative predictive value = true negatives/(true negatives + false negatives). Fisher’s exact test or the *χ*^2^ test was used to examine the differences between patients with and without LNM/high-volume LNM. Multivariate models were used to determine the risk factors for LNM/high-volume LNMs; *p* value < 0.05 was considered to indicate statistical significance.

## Results

### Clinicopathological backgrounds

A total of 2073 PTC patients were included in the study for analysis (male: 490, female: 1583). Of all the patients, 936 (45.2%) patients had LNM, and 254 (12.3%) patients had high-volume LNMs. A total of 1414 cases were papillary thyroid microcarcinoma (PTMC), 513 (36.3%) patients had LNM, and 96 (6.8%) patients had high-volume LNMs (Table 1).

**Table 1:** Patient demographics

Item	PTC	PTMC
Sex		
Female	1583 (76.4)	1098 (77.7)
Male	490 (23.6)	316 (22.3)
Age		
≤55	1755 (84.7)	1200 (84.9)
>55	318 (15.3)	214 (15.1)
BMI		
18.5	55 (2.7)	37 (2.6)
<18.5–24	970 (46.8)	664 (47.0)
<24–28	755 (36.4)	511 (36.1)
28~	293 (14.1)	202 (14.3)
Tumor diameter^a^		
≤1 cm	1414 (68.2)	–
≤0.5	–	315 (22.3)
>0.5–1.0	–	1099 (77.7)
>1.0–2.0	499 (24.1)	–
>2.0	160 (7.7)	–
usLNM		
Yes	339 (16.4)	157 (11.1)
No	1734 (83.6)	1257 (88.9)
Operation		
Hemithyroidectomy + LND	410 (19.8)	342 (24.1)
Total thyroidectomy + LND	1663 (80.2)	1072 (75.9)
Multilfocality		
Yes	731 (35.3)	457 (32.3)
No	1342 (64.7)	957 (67.7)
Capsule invasion		
Yes	643 (31.0)	320 (22.6)
No	1430 (69.0)	1094 (77.4)
Chronic lymphocytic thyroiditis		
Yes	509 (24.6)	337 (23.8)
No	1564 (75.4)	1077 (76.2)
LNM		
Yes	936 (45.2)	513 (36.3)
No	1137 (54.8)	901 (63.7)
hvLNMs		
Yes	254 (12.3)	96 (6.8)
No	1819 (87.7)	1318 (93.2)

*BMI* body mass index, *usLNM* lymph node involvement on ultrasound, *LND* lymph node dissection, *LNM* lymph node metastasis, *hvLNMs* high-volume lymph node metastases

^a^Diameter of the largest lesion in multifocal tumors

### Efficacy of preoperative ultrasound for LNM and high-volume LNMs

A total of 339 (16.4%) patients showed lymph node involvement on preoperative ultrasound, of whom 261 (77.0%) had LNM confirmed by postoperative pathology; a total of 1734 (83.6%) patients showed no lymph node involvement on preoperative ultrasound, of whom 1137 (65.6%) had no LNM confirmed by postoperative pathology. Among the PTMC patients, a total of 157 (11.1%) showed lymph node involvement on preoperative ultrasound, of whom 101 (64.3%) had LNM confirmed by postoperative pathology; a total of 1257 (88.9%) patients showed no lymph node involvement on preoperative ultrasound, of whom 845 (67.2%) had no LNM confirmed by postoperative pathology. The sensitivity of preoperative ultrasound for detecting LNM was 27.9% (261/936), the specificity was 93.1% (1059/1137), the negative predictive value (NPV) was 61.1% (1059/1734), and the accuracy rate was 63.7% (1320/2073) (Table 2).

**Table 2:** Accuracy evaluation of ultrasound prediction of lymph node metastases

Item	PTMC *n* = 1414		Non-PTMC *n* = 659		PTC *n* = 2073	
	LNM	hvLNMs	LNM	hvLNMs	LNM	hvLNMs
Sensitivity	19.7%(101/513)	57.3% (55/96)	37.8% (160/423)	67.7% (107/158)	27.9% (261/936)	63.8% (162/254)
Specificity	93.8% (845/901)	92.3% (1216/1318)	90.7% (214/236)	85.0% (426/501)	93.1% (1059/1137)	90.3% (1642/1819)
PPV	64.3% (101/157)	35.0% (55/157)	87.9% (160/182)	58.8% (107/182)	77.0% (261/339)	47.8% (162/339)
NPV	67.2% (845/1257)	96.7% (1216/1257)	44.9% (214/477)	89.3% (426/477)	61.1% (1059/1734)	94.7% (1642/1734)
Accuracy	66.9%(946/1414)	89.9% (1271/1414)	56.8% (374/659)	80.9% (533/659)	63.7% (1320/2073)	87.0% (1804/2073)

*LNM* lymph node metastasis, *hvLNMs* high-volume lymph node metastases, *PPV* positive predictive value, *NPV* negative predictive value

Among 339 (16.4%) PTC patients with lymph node involvement on preoperative ultrasound, 162 (47.8%) patients had confirmed high-volume LNMs by postoperative pathology; among 1734 (83.6%) patients with no lymph node involvement on preoperative ultrasound, 1642 (94.7%) patients had confirmed absence of high-volume LNMs by postoperative pathology. Among 157 (11.1%) PTMC patients with lymph node involvement on preoperative ultrasound, 55 (35.0%) patients had confirmed high-volume LNMs by postoperative pathology; among 1257 (88.9%) patients with no lymph node involvement on preoperative ultrasound, 1216 (96.7%) patients had confirmed absence of high-volume LNMs by postoperative pathology. The sensitivity of preoperative ultrasound for predicting high-volume LNMs was 63.8% (162/254), the specificity was 90.3% (1642/1819), the negative predictive value (NPV) was 94.7% (1642/1734), and the accuracy rate was 87.0% (1804/2073) (Table 2).

### Univariate and multivariate analyses for LNM

Univariate analysis showed that compared with control group (patients without LNM), LNM were more common in male sex (58.4% vs. 41.1%, *p* < 0.001), age < 55 years old (47.0% vs. 34.9%, *p* < 0.001), tumor diameter > 1 cm (64.2% vs. 36.3%, *p* < 0.001) and usLNM (+) patients (77.0% vs. 38.9%, *p *< 0.001) (Table 3). Multivariate analysis showed that male sex (OR = 1.869, 95% CI 1.499–2.331, *p *< 0.001), tumor diameter > 1.0 cm (OR = 2.203, 95% CI 1.892–2.565, *p *< 0.001) and usLNM (+) (OR = 3.978, 95% CI 2.999–5.275, *p *< 0.001) were independent risk factors for LNM. In contrast, age > 55 years old (OR = 0.605, 95% CI 0.462–0.791, *p *< 0.001) was an independent protective factor for LNM (Table 4).

**Table 3:** Univariate analysis of risk factors for pathological LNM and hvLNMs

Item	LNM (−)	LNM (+)	*P* value	hvLNMs (−)	hvLNMs (+)	*P* value
	*n* = 1137 (%)	*n* = 936 (%)		*n* = 1819 (%)	*n* = 254 (%)	
Sex			<0.001			<0.001
Female	933 (58.9)	650 (41.1)		1428 (90.2)	155 (9.8)	
Male	204 (41.6)	286 (58.4)		391 (79.8)	99 (20.2)	
Age			<0.001			0.04
≤55	930 (53.0)	825 (47.0)		1529 (87.1)	226 (12.9)	
>55	207 (65.1)	111 (34.9)		290 (91.2)	28 (8.8)	
BMI			0.950			0.685
~18.5	32 (58.2)	23 (41.8)		50 (90.9)	5 (9.1)	
<18.5–24	534 (55.1)	436 (44.9)		851 (87.7)	119 (12.3)	
<24–28	410 (54.3)	345 (45.7)		666 (88.2)	89 (11.8)	
28~	161 (54.9)	132 (45.2)		252 (86.0)	41 (14.0)	
Tumor diameter^a^			<0.001			<0.001
≤1 cm	901 (63.7)	513 (36.3)		1318 (93.2)	96 (6.8)	
>1 cm	236 (35.8)	423 (64.2)		501 (76.0)	158 (24.0)	
usLNM			<0.001			<0.001
Yes	78 (23.0)	261 (77.0)		177 (52.2)	162 (47.8)	
No	1059 (61.1)	675 (38.9)		1642 (94.7)	92 (5.3)	

*BMI* body mass index, *usLNM* lymph node involvement on ultrasound, *LNM* lymph node metastasis, *hvLNMs* high-volume lymph node metastases

^a^Diameter of the largest lesion in multifocal tumors

**Table 4:** Multivariate analysis of risk factors for pathological LNM and hvLNMs

Item	OR	95% CI	*P* value
LNM			
usLNM (+)	3.978	2.999–5.275	<0.001
Tumor diameter^a^ > 1 cm	2.203	1.892–2.565	<0.001
Male	1.869	1.499–2.331	<0.001
BMI ≥ 24	1.015	0.842–1.225	0.873
Age > 55	0.605	0.462–0.791	<0.001
hvLNMs			
usLNM (+)	12.553	9.181–17.165	<0.001
Tumor diameter^a^ > 1 cm	2.304	1.889–2.811	<0.001
Male	2.108	1.517–2.929	<0.001
BMI ≥ 24	1.034	0.757–1.414	0.832
Age > 55	0.728	0.449–1.181	0.119

*OR* odds ratio, *CI* confidence interval, *BMI* body mass index, *usLNM* lymph node involvement on ultrasound, *LNM* lymph node metastasis, *hvLNMs* high-volume lymph node metastases

^a^Diameter of the largest lesion in multifocal tumors

Univariate analysis was used to evaluate the differences between patients with (254 cases) and without high-volume LNMs (1819 cases). Results showed that compared with control group (patients without high-volume LNMs), high-volume LNMs were more common in male sex (20.2% vs. 9.8%, *p* < 0.001), age ≤ 55 years old (12.9% vs. 8.8%, *p* < 0.001), tumor diameter > 1 cm (24.0% vs. 6.8%, *p *< 0.001) and usLNM (+) patients (47.8% vs. 5.3%, *p *< 0.001) (Table 3). Multivariate analysis showed that male sex (OR = 2.108, 95% CI 1.517–2.929, *p *< 0.001), tumor diameter > 1.0 cm (OR = 2.304, 95% CI 1.889–2.811, *p *< 0.001) and usLNM (+) (OR = 12.553, 95% CI 9.181–17.165, *p *< 0.001) were independent risk factors for high-volume LNMs (Table 4).

### Univariate and multivariate analyses for usLNM

The number of patients in group A and B was 1251 and 822, respectively. There were no significant differences in clinicopathologic features between the two groups (Supplementary Table). Univariate analysis showed that usLNM was more common in male sex (22.4% vs. 14.5%, *p *< 0.001), age ≤ 55 years old (17.2% vs. 11.9%, *p* = 0.021) and tumor diameter > 1 cm patients (27.6% vs. 11.1%, *p *< 0.001) (Table 5). Multivariate analysis showed that male sex (OR = 1.642, 95% CI 1.264–2.133, *p *< 0.001) and tumor diameter > 1 cm (OR = 3.036, 95% CI 2.389–3.859, *p *< 0.001) were independent risk factors for usLNM. In contrast, age > 55 years old (OR = 0.646, 95% CI 0.446–0.935, *p* = 0.02) was an independent protective factor for usLNM, and a skilled sonographer (OR = 1.121, 95% CI 0.879–1.429, *p *= 0.358) was not significantly associated with usLNM (Table 6).

**Table 5:** Univariate analysis of risk factors for usLNM

Item	usLNM (−)	usLNM (+)	*P* value
	*n* = 1734 (%)	*n* = 339 (%)	
Sex			<0.001
Female	1354 (85.5)	155 (14.5)	
Male	391 (77.6)	99 (22.4)	
Age			0.021
≤55	1454 (82.8)	301 (17.2)	
>55	280 (88.1)	38 (11.9)	
BMI			0.844
~18.5	48 (87.3)	7 (12.7)	
<18.5–24	806 (83.1)	164 (16.9)	
<24–28	634 (84.0)	121 (16.0)	
28–	246 (84.0)	47 (16.0)	
Tumor diameter^a^			<0.001
≤1 cm	1257 (88.9)	157 (11.1)	
>1 cm	477 (72.4)	182 (27.6)	
Sonographer			0.363
Group A	1054 (84.3)	197 (15.7)	
Group B	680 (82.7)	142 (17.3)	

*BMI* body mass index, *usLNM* lymph node involvement on ultrasound

^a^Diameter of the largest lesion in multifocal tumors; A general sonographer; B skilled sonographer

**Table 6:** Multivariate analysis of risk factors for usLNM

Item	OR	95% CI	*P* value
Male	1.642	1.264–2.133	<0.001
Age > 55	0.646	0.446–0.935	0.02
Tumor diameter^a^ > 1 cm	3.036	2.389–3.859	<0.001
BMI ≥ 24	0.929	0.715–1.208	0.583
Sonographer B	1.121	0.879–1.429	0.358

*OR* odds ratio, *CI* confidence interval, *BMI* body mass index, *usLNM* lymph node involvement on ultrasound

^a^Diameter of the largest lesion in multifocal tumors; B skilled sonographer

## Discussion

Our study showed that the sensitivity of LNM detection for preoperative ultrasound was relatively low: 27.9% for PTC and 19.7% for PTMC. However, the sensitivity of high-volume LNMs detection was significantly increased (63.8%); furthermore, the NPV could reach 94.7% for PTC and 96.7% for PTMC. Obviously, the impact of the diagnostic efficacy of preoperative ultrasound on the clinical diagnosis and treatment of PTC is worthy of deep discussion.

An increase in the incidence of thyroid cancer, especially PTC, has been reported in several countries [12, 13], including China [14], over the past several decades. Although the overall survival of PTC patients is better than that of patients with other cancers, regional LNMs are frequently observed in PTC [15]. The lymph node status affects decision-making in terms of treatment and prognosis in patients with PTC [16]. Ultrasound has been the first choice of imaging modality for the preoperative assessment of LNM. However, the sensitivity of ultrasound for detecting LNM of the central neck was only 33%, and the specificity was 93%; for the lateral compartment, the sensitivity and specificity were 70% and 84%, respectively [17]. Preoperative ultrasound only detects half of the lymph nodes found during surgery [18]. Our data also showed that preoperative ultrasound had poor diagnostic accuracy for assessing cervical LNM in PTC, and the sensitivity, specificity and accuracy of preoperative ultrasound for cervical LNM were 27.9%, 93.1% and 63.7%, respectively. In the face of this diagnostic efficacy for LNM, are the results of ultrasound still worth trusting?

The failure of preoperative ultrasound to detect metastatic lesions may be common. However, these lymph node involvements that cannot be showed on ultrasound may be representative of fewer and smaller lesions and may not have a significant influence on treatment outcomes. In regions routinely performing prophylactic LND, the LNM rate of PTMC can reach 30–40% [19, 20], but it has been reported to be only 3.2% in regions without routine prophylactic LND. However, the postoperative recurrence and reoperation rate of the two groups did not seem to have an obvious difference [20, 21]. The 2015 ATA guidelines emphasized the impact of the size and number of metastatic lesions on prognosis. The recurrence rate of patients with high-volume LNMs (>5 metastatic lymph nodes) was 19%, which was significantly higher than the recurrence rate of 2–4% in cN0pN1 patients (<5 metastatic lymph nodes) [8]. These patients with high-volume LNMs should be identified and provided treatment cautiously, as their treatment outcomes have a great impact on the prognosis of disease. Based on our results, when lymph node involvement was detected by ultrasound, the rate of postoperative high-volume LNMs was 47.8%; when lymph node involvement was not detected, the rate of postoperative high-volume LNMs was 5.3%. The sensitivity, specificity and accuracy of the diagnosis were 63.8%, 90.3% and 87.0%, respectively. These findings indicate that lymph node involvement found by ultrasound has great value for predicting postoperative high-volume LNMs. Furthermore, when no lymph node involvement was detected by preoperative ultrasound, the negative predictive value for high-volume LNMs was 94.7%, which was much higher for PTMC (96.7%). The results suggest that the possibility of high-volume LNMs is very low for patients without lymph node involvement on preoperative ultrasound, and less aggressive treatment (active surveillance or treatment without prophylactic LND) may not bring poor outcomes for these patients.

Our results showed that among the clinical factors that may affect the prediction of high-volume LNMs, male sex (20.2% vs. 9.8%, *p *< 0.001), age ≤ 55 years old (12.9% vs. 8.8%, *p *< 0.001) and tumor diameter > 1 cm (24.0% vs. 6.8%, *p *< 0.001) were risk factors for high-volume LNMs, results that were similar to those of previous studies [22, 23]. Nevertheless, the risk factor for high-volume LNMs, which was more important than these known clinical risk factors, was lymph node involvement detected by preoperative ultrasound with an OR of 12.553. Ultrasound evaluation is an operator-dependent method [24]. Surgeons should be particularly vigilant regarding whether the experience of the sonographer will affect the ultrasound results, especially in the evaluation of cervical lymph node involvement, which has good predictive value for high-volume LNMs. Our results showed that the proportion of LNM detected by skilled and general sonographers was 17.3% and 15.7% (*p *= 0.363) in the two groups with similar conditions, respectively (Supplementary Table). In the multivariate analysis, a skilled sonographer was not an independent risk factor for detecting LNM by ultrasound (OR = 1.121, 95% CI 0.879–1.429, *p *= 0.358). However, this study was not a standard diagnostic test; therefore, a standard diagnostic test should be conducted to clarify the impact of sonographer experience on ultrasound results. Moreover, there are needs to expand the population and conduct a multi-center prospective study to evaluate the value of ultrasound in predicting high-volume lymph node metastases for the results just from a retrospective study in one center.

## Conclusion

Although preoperative ultrasound has poor sensitivity for detecting LNM, lymph node involvement found by ultrasound has great predictive value for high-volume LNMs; in particular, the NPV is very high for patients without lymph node involvement on preoperative ultrasound. Therefore, it is acceptable to adopt less aggressive treatment strategies for these patients. Moreover, the use of preoperative ultrasound for predicting high-volume lymph node metastases does not appear to be influenced by the experience of the sonographer.

## Electronic supplementary material

Below is the link to the electronic supplementary material.

Supplementary material 1 (DOCX 14 kb)

## Abbreviations

LNM: Lymph node metastasis
LNMs: Lymph node metastases
PTC: Papillary thyroid carcinoma
PTMC: Papillary thyroid microcarcinoma
LND: Lymph node dissection
PPV: Positive predictive value
NPV: Negative predictive value
usLNM: Lymph node involvement on ultrasound
