# Supplementary material for: Ultrasonography for the Prediction of High-Volume Lymph Node Metastases in Papillary Thyroid Carcinoma: Should Surgeons Believe Ultrasound Results?
Source: World J Surg. 2020 Sep 11;44(12):4142–8. doi: 10.1007/s00268-020-05755-0 (PMC7599182; doi:10.1007/s00268-020-05755-0)
Supplement: Supplementary file 1 — Supplementary material 1 (DOCX 14 kb) [file 268_2020_5755_MOESM1_ESM.docx]

| Table Clinical and pathological features of patients in Group A/B | | | |
| --- | --- | --- | --- |
| Item | Group A  N=1251（%） | Group B  N=822（%） | P-value |
| Sex |  |  | 0.526 |
| Female | 949 (75.9) | 634 (77.1) |  |
| Male | 302 (24.1) | 188 (22.9) |  |
| Age |  |  | 0.263 |
| ≤ 55 | 1050 (83.9) | 462 (85.8) |  |
| > 55 | 201 (16.1) | 360 (14.2) |  |
| BMI |  |  | 0.753 |
| ~18.5 | 32 (2.6) | 23 (2.8) |  |
| ＜18.5~24 | 580 (46.3) | 390 (47.4) |  |
| ＜24~28 | 454 (36.3) | 301 (26.6) |  |
| 28~ | 185 (14.8) | 108 (13.1) |  |
| Tumor diameter ^a^ |  |  | 0.861 |
| ≤1cm | 855 (68.3) | 559 (68.0) |  |
| ≤0.5cm | 194 (15.5) | 121 (14.7) |  |
| ＞0.5~1.0 cm | 661 (52.8) | 438 (53.3) |  |
| ＞1.0~2.0 cm | 296 (23.7) | 203 (24.7) |  |
| ＞2.0 cm | 100 (8.0) | 60 (7.3) |  |
| Multilfocality |  |  | 0.481 |
| Yes | 449 (35.9) | 282 (34.3) |  |
| No | 802 (64.1) | 540 (65.7) |  |
| Capsule invasion |  |  | 0.734 |
| Yes | 392 (31.3) | 251 (30.5) |  |
| No | 859 (68.7) | 571 (69.5) |  |
| LNM |  |  | 0.558 |
| Yes | 558 (44.6) | 378 (46.0) |  |
| No | 693 (55.4) | 444 (54.0) |  |
| hvLNMs |  |  | 0.218 |
| Yes | 144 (11.5) | 110 (13.4) |  |
| No | 1107 (88.5) | 712 (86.6) |  |

Abbreviation: BMI body mass index; LNM lymph node metastasis; hvLNMs high-volume lymph node metastases; ^a^ diameter of the largest lesion in multifocal tumors.
